# Hyperemesis Gravidarum Complicated by Wernicke’s Encephalopathy: A Case Report

**DOI:** 10.7759/cureus.24009

**Published:** 2022-04-10

**Authors:** Natalie Punal, Supritha Prasad, Afsara Haque, Justin Lei, Gaia Santiago

**Affiliations:** 1 Department of Internal Medicine, University of Illinois at Chicago, Chicago, USA; 2 Department of Medicine, University of Illinois at Chicago, Chicago, USA

**Keywords:** altered mental status, opthalmoplegia, thiamine deficiency, hyperemesis gravidarum, wernicke’s encephalopathy (we)

## Abstract

Wernicke’s encephalopathy (WE) is a rare neurologic disease caused by a deficiency in thiamine (B1). It is characterized by features of altered mental status, cerebellar dysfunction, and ophthalmoplegia. Most often, cases are attributed to long-term alcohol use; however, rarer causes have been described in the literature. In this article, we describe a case of WE caused by hyperemesis gravidarum in a 19-year-old female with no known medical history.

## Introduction

Wernicke’s encephalopathy (WE) is a rare neurologic disorder caused by a deficiency in thiamine (B1) that is classically characterized by altered mental status, ataxia, and ophthalmoplegia. Thiamine is a cofactor in both the citric acid cycle and pentose phosphate pathway, serving a vital role in cellular metabolism, aerobic respiration, and anabolic processing [1]. Deficiency in thiamine leads to decreased levels of alpha-ketoglutarate, acetate, citrate, and acetylcholine. An inability to progress through these biochemical pathways results in the accumulation of byproducts, such as lactate and pyruvate, ultimately resulting in cytotoxic edema and cellular death [1]. Thiamine is also used in the synthesis of neurotransmitters, proteins, and complex sugars that are essential to healthy brain function [2]. Most cases of WE in developed countries occur secondary to chronic alcohol use; however, other causes have been described in the literature.

## Case presentation

A 19-year-old gravida 2 para 1 female at 17 weeks of gestation was sent to the emergency department for evaluation of altered mental status from an abortion clinic. Her history was limited at the time of initial presentation secondary to confusion. Per a review of outside hospital charts, the patient had presented to an emergency department two times prior for severe vomiting, was found to have severe electrolyte abnormalities, and was given fluid resuscitation. She was then referred to the university hospital abortion clinic by her outside obstetrician for unclear reasons.

Upon arrival at the emergency department, our patient was found to be confused, only able to answer simple “yes or no” questions. Initial vital signs were notable for heart rate in the 120s, blood pressure of 90s/50s, normal respiratory rate, and oxygenation of >95% on room air. Laboratory results demonstrated severe metabolic derangements with potassium of 2.7 mmol/L, anion gap of 20, creatinine of 2.37 mg/dL, and lactic acid level of 7.2 mmol/L. A comprehensive list of initial lab values is included in Table 1. A physical examination was significant for altered mental status with the patient only being alert and oriented to self, along with dry and cracked mucus membranes. A neurologic examination was significant for vertical pendular nystagmus in all directions of gaze with dysmetria in bilateral upper extremities. Fluid resuscitation and thiamine infusion were initiated with concern for WE.

**Table 1:** Relevant laboratory values. Laboratory values on admission and 12 hours post-admission.

Labs	Admission	12 hours after admission	Reference range
Sodium	132 mmol/L	138 mmol/L	135–145 mmol/L
Potassium	2.7 mmol/L	3.1 mmol/L	3.5–4.2 mmol/L
Chloride	82 mmol/L	101 mmol/L	98–108 mmol/L
Bicarbonate	26 mmol/L	28 mmol/L	24–32 mmol/L
Blood urea nitrogen	32 mg/dL	20 mg/dL	6–20 mg/dL
Creatinine	2.37 mg/dL	1.01 mg/dL	0.4–1.2 mg/dL
Lactic acid	7.2 mmol/L	1.6 mmol/L	0–2.2 mmol/L
Anion gap	20	9	0–13
pH (mixed venous)	7.40	7.43	7.33–7.42
CO_2_ (mixed venous)	41 mmHg	42 mmHg	40–52 mmHg

**Figure 1:**
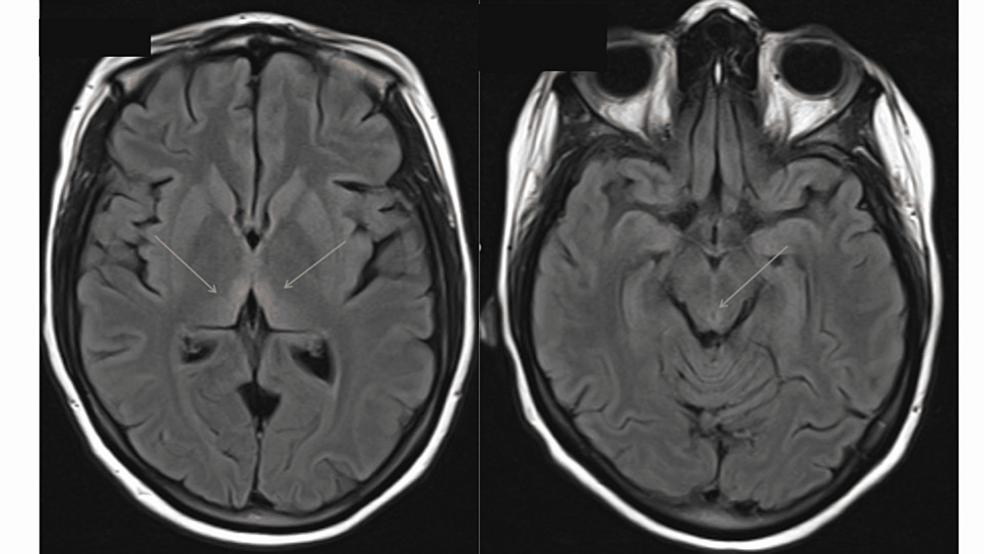
MRI of the brain with and without contrast. MRI brain showing T2 flair hyperintensities in dorsomedial thalamus (left) and periaqueductal region (right), consistent with the presumed diagnosis of Wernicke’s encephalopathy. MRI: magnetic resonance imaging

Volume and electrolyte repletion were continued upon admission to the general medicine team; however, vertical nystagmus on examination raised suspicion for cerebellar stroke, and thus emergent Magnetic resonance imaging (MRI) imaging of the brain was performed. The MRI brain demonstrated prominent increased T2 signal abnormality along the medial aspect of the thalami bilaterally without depressed apparent diffusion coefficient (Figure 1), consistent with the presumed diagnosis of WE.

After 12 hours of fluid resuscitation, electrolyte repletion and intravenous thiamine, our patient’s mental status improved significantly, and she was able to converse with the primary team to provide additional history. She stated she had lost approximately 30 pounds over the past month with four to five episodes of emesis per day. She denied any vitamin supplementation at the time. She had presented to an outside emergency department around two to three times, was given fluids and electrolytes, and sent home.

She was continued on thiamine 500 mg intravenously (IV) every eight hours for three days, transitioned to IV 250 mg thiamine every eight hours for five days, and finally placed on oral 100 mg thiamine daily indefinitely per the neurology team’s recommendations. Although her vertical nystagmus improved, she continued to have severe weakness and poor coordination, making it difficult to stand without assistance.

On hospital day two, she was evaluated by the physical therapists and found to have significant weakness in bilateral lower extremities with ataxia. She continued physical and occupational therapy, and on hospital day 10, she showed significant improvement in balance and strength. The physical exam was notable for nystagmus but was improved from the initial presentation. After discussion with obstetrics, our patient elected to continue her pregnancy with fetal ultrasound showing no abnormalities.

On hospital day 23, she was discharged to her brother’s house for continued therapy. At the time of discharge, she required a walker to move independently. She was continued on thiamine, folate, and multivitamin therapy indefinitely upon discharge.

## Discussion

Nausea and vomiting are among the most common complications of pregnancy. It is estimated that close to 70% of women develop some form of nausea and vomiting during pregnancy (NVP) [3]. At the severe end of this spectrum is hyperemesis gravidarum (HG). The American College of Obstetrics and Gynecology (ACOG) guidelines for NVP does not contain a single accepted definition of HG; therefore, it is widely considered a diagnosis of exclusion. The most accepted definition is a pregnant female with severe vomiting causing malnutrition (ketonuria or weight loss) [4]. Little is known about the pathophysiology of HG, and previous studies aimed to discover if b-hCG levels were a predisposing factor. However, no true correlation has ever been established between b-hCG and HG. More recent studies suggest a multifactorial cause with genetic predisposition becoming a more prominent risk factor [5]. Regardless of the cause, HG poses a great risk to both the mother and the fetus. A retrospective study done between 1988 and 2002 showed babies born to women with HG have an increased risk of low birth weight, preterm delivery, and a five-minute Apgar score of less than 7 [6]. More recent studies have shown that babies born to mothers with HG also have an increased risk of developing psychological and behavior disorders later in life [7]. HG also poses a great risk to the mother, some of the most common complications are esophageal injury, psychological distress, and nutritional deficiencies [8]. Although death due to HG is rare, it is most often associated with WE [8].

WE is most frequently cited in the literature in association with chronic alcohol use; however, other etiologies have been described. In a post-mortem autopsy study, approximately 23% of cases of WE found occurred in non-alcoholics [9]. More interestingly, of all the cases of WE found (52 of 6,964 autopsies), only four cases were diagnosed before death, all of which were among alcoholics [9]. These findings suggest that WE has been underdiagnosed and underrecognized in the severely malnourished population.

WE is a clinical diagnosis that can be difficult to observe in non-alcoholics. While ophthalmoplegia, mental status changes, and unsteadiness of stance and gait are considered the classic triad of symptoms, these symptoms are only present in 16% of cases [4]. The criteria for clinical diagnosis of WE have been expanded to improve premortem diagnosis, including dietary deficiencies, oculomotor abnormalities, cerebellar dysfunction, and either an altered mental state or mild memory impairment [10]. The presence of two or more of the aforementioned is highly suggestive of WE. Furthermore, there is no imaging study or confirmatory test to diagnose WE. The most distinctive radiologic findings are cytotoxic and vasogenic edema in the periphery of the third ventricle, periaqueductal area, mammillary bodies, and midbrain, which are most commonly seen on T2-weighted MRI [11]. Our patient had all four symptoms of WE in addition to T2-weighted MRI images suggestive of WE.

Treatment is thiamine supplementation; however, the optimal dosage and timeframe of therapy have not been well established. Typically, initiation of thiamine intravenously is advised with the most commonly used regimen of 500 mg three times a day for three to five days [12]. The duration of therapy is not clinically standardized either. The Royal College of Physicians recommends intravenous 500 mg three times a day for two to three days, intravenous 250 mg three times a day for two to three days, followed by indefinite oral therapy [13]. Our patient was treated with the latter regimen with noticeable improvement from the initial presentation.

## Conclusions

Our case represents a case of nutritional deficiency leading to WE in an unsuspecting population. It is critical that we identify individuals at risk before serious consequences such as permanent neurologic damage or even death. Given the known detriment of thiamine deficiency with only a few known adverse effects of thiamine therapy, it is important we not only keep WE in our differential diagnosis but initiate early treatment, especially for anyone with severe malnutrition presenting with neurologic symptoms.
